# Molecular Characterization of Pestiviruses in Fetal Bovine Sera Originating From Argentina: Evidence of Circulation of HoBi-Like Viruses

**DOI:** 10.3389/fvets.2019.00359

**Published:** 2019-10-16

**Authors:** Andrea Pecora, Maria Sol Perez Aguirreburualde, Julia Francis Ridpath, María José Dus Santos

**Affiliations:** ^1^Center of Veterinary Sciences and Agronomic Investigations, Institute of Virology, INTA, Buenos Aires, Argentina; ^2^National Council for Scientific and Technical Research (CONICET), Buenos Aires, Argentina; ^3^Center for Animal Health and Food Safety, University of Minnesota, Minneapolis, MN, United States; ^4^Ridpath Consulting, LLC, Gilbert, IA, United States

**Keywords:** adventitious agent, HoBi-like, pestivirus, diagnostic techniques, survey

## Abstract

Serological evidence suggests that HoBi-like viruses, an emerging species within the Pestivirus genus of the Flaviviridae family, are in circulation in Argentina. While HoBi-like viruses were first isolated from Brazilian fetal bovine serum (FBS), no survey of Argentine FBS has been conducted. Therefore, 124 local samples of non-irradiated FBS originating from Argentina were surveyed for the presence of pestiviruses using RT-PCR. Amplicons from pestivirus positive samples were genotyped. Four samples were positive for HoBi virus-specific RT-PCR, while the BVDV-positive samples (*n* = 45) were classified as BVDV-1b (82.2%), BVDV-1a (13.3%), and BVDV-2 (4.5%). Virus isolation and serological profile assessment were performed for the four HoBi-positive FBS lots. These results confirm the circulation of HoBi-like virus in some regions of the Argentinean territory, highlighting the need to review the diagnostic techniques currently used in the clinical cases suspected of BVDV and in contamination control protocols for adventitious agents in cells and biotechnological products.

## Introduction

Pestiviruses belong to the *Flaviviridae* family and are enveloped, positive-sense, single-stranded RNA viruses. The pestivirus genome consists of an RNA molecule of about 12,300 nucleotides, containing a single open reading frame that codes for a single polyprotein precursor of 3,900 amino acids. The number of members of the pestivirus genus is currently under review as the incorporation of new putative species has been proposed. Initially, the four recognized species were Pestivirus A, Pestivirus B, and Pestivirus C (Bovine Viral Diarrhea Virus, Border Disease Virus, and Classical Swine Fever Virus, respectively), but now the number of species may reach 11, designed as “pestivirus A” to “pestivirus K.” Within the “new species” of pestiviruses, “pestivirus H,” also known as HoBi-like virus, BVDV-3 or atypical ruminant pestivirus, was one of the most frequently isolated (1).

The array of clinical presentations that collectively fall under that title bovine viral diarrhea (BVD) are caused by three species within the pestivirus genus, BVDV-1, BVDV-2, and HoBi-like virus (2). Since the first identification in Brazilian Fetal Bovine Serum (FBS) in 2004, several HoBi-like virus isolates have been described in Brazil, Europe, and Asia (3–7). This viral agent is a common contaminant in biological products such as FBS; so much so that 40% of all HoBi-like pestivirus strains identified worldwide, to date, were recovered from FBS or cell culture samples (8, 9). Detection of HoBi-like virus is currently a major concern in the area of diagnosis, because many tests available for detecting the causative agents of BVD were designed specifically to detect BVDV-1 and BVDV-2 and may not reliably detect HoBi-like virus.

FBS comes from the blood drawn from bovine fetuses via a closed system of collection at the slaughterhouses. FBS is frequently used to supplement cell cultures employed in basic biomedical research and the development of diagnostic tests. In addition, FBS is used extensively in the biotechnology industry for the production of traditional viral vaccines, recombinant proteins, and biotherapeutics. This is because it is a rich source of essential growth factors, amino acids, proteins, vitamins, carbohydrates, lipids, hormones, growth factors, minerals, and trace elements needed for cell growth stimulation. Originally, FBS was used rather than serum from adult animals because it was thought that blood collected from fetuses was less likely to be contaminated with infectious agents than blood collected from adult animals. However, we now know that cows can be infected with pathogens during pregnancy and can pass these pathogens through the placenta to fetuses, which are the actual sources of FBS (10). This results in FBS becoming contaminated with pathogens such as viruses. These pathogens play a role as adventitious agents that are accidentally introduced through FBS use during the manufacturing of biological products. Unlike microbial contamination (fungi, yeast, and bacteria), the detection of mycoplasma and non-cytopathic (ncp) viruses is more difficult since their effects on cultured cells cannot be detected by routine microscopy. The potential of viruses to cause a silent infection in a cell culture must be addressed, since contaminating viruses can affect research and diagnostic outcomes. In addition, viral contamination of cells and biological products can have potentially dramatic epidemiological consequences, through the accidental introduction of pathogens into human or animal susceptible populations (11). Although downstream processing may be able to eliminate or inactivate certain viruses, others may be resistant to these procedures, highlighting the need to update regulatory risk evaluation and to develop more stringent and detailed requirements (https://www.who.int/bloodproducts).

Pestiviruses are one of the most common viral contaminants found in FBS (10). However, the risk of having an infectious virus in the FBS can be reduced by gamma-irradiation (12). Although the commercialization of gamma-irradiated FBS is recommended, some companies market it without the irradiation process and this could lead to silent contamination of products in the research and industrial sector. Further, gamma-irradiation may not be 100% effective under all conditions (13). Besides Brazil, the epidemiology of Hobi-like virus in South America is unknown (14). In Argentina, we have previously reported serologic evidence of natural circulation of this agent in bubaline populations of the northeast region of Argentina, bordering Brazil. However, live virus was not isolated from samples collected for that survey (15). Moreover, the most recent survey of the BVDV genotypes and subgenotypes affecting the bovine populations of Argentina was generated from viruses isolated before 2010, using PCR protocols that do not detect HoBi-like viruses (16).

The analysis of FBS-samples originating from Pampean region (central eastern of Argentina) would allow an estimation of the frequency of genotypes and subgenotypes of pestiviruses in local herds. Further, analyzing FBS samples allows a sampling strategy with a wider coverage and simpler accessibility than traditional sampling strategies of recruiting and testing herds.

## Materials and Methods

### Fetal Bovine Serum Samples

**Figure 1:**
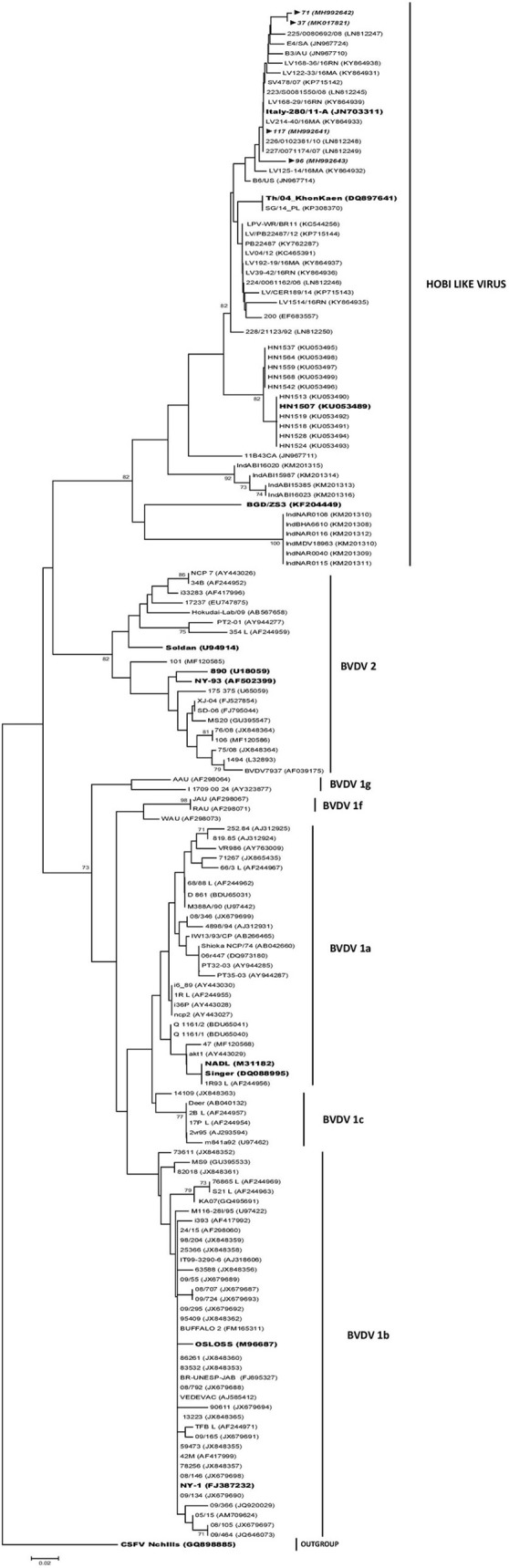
Geographic location of the slaughterhouses.

For the purposes of this study, 124 samples of non-irradiated fetal bovine sera batches, collected between 2014 and 2106, were obtained from two FBS manufacturers in Argentina. Each sample corresponded to a pool of 20 fetuses. Aliquots of 1.0 ml of each serum were placed in nuclease-free microcentrifuge tubes and stored at −70°C prior to analysis. Fetuses were collected from 30 slaughterhouses located in Buenos Aires province (provider A, 121 samples) and three samples corresponded to two slaughterhouses located in Córdoba and Santa Fe provinces (provider B, three samples; Figure 1). These three provinces accumulate 55.3% of the national cattle herd (Buenos Aires 35%; Santa Fe 11.3%; Cordoba 9%; http://www.senasa.gob.ar). All the slaughterhouses processed both dairy and feedlot herds. The samples analyzed were regarded as “semi-processed products” because they were not irradiated. Some of those lots were also tested after irradiation (corresponding to “final products”), to check the effectiveness of the Gamma-irradiation process. The dynamic irradiation was performed in a local company (IONICS, S.A.) at 25 Kilograys (KGrays).

### RNA Purification and Reverse Transcription

Viral RNA was extracted directly from 200 μl of FBS samples using a commercial kit (Roche High pure Viral RNA kit) according to the manufacturer's recommendations. The positive controls (BVDV Singer strain and HoBi 83/10 CP (Decaro N, Department of Veterinary Medicine of Bari, Italy) were virus grown in cultured cells and diluted in FBS to a final titer of 10^2^ tissue culture infective dose (TCID)/ml. Aliquots of pestivirus negative FBS, minimal essential media (MEM), and supernatants of mock infected Madin-Darby bovine kidney (MDBK) cells were used as negative controls. Viral RNA was extracted from 200 μl of each control and was processed as the FBS samples. Reverse transcription was carried out in a 25 μl volume using 500 ηg of RNA and 0.5 μg of random hexanucleotide primers (Biodynamics, SRL). Two hundred units of M-MLV (Promega) and 20 units RNAsin ribonuclease inhibitor (Promega) were used with 5 μl of 5X RT Buffer (50 mM Tris–HCl, 75 mM KCl, 3 mM MgCl_2_, and 10 mM DTT), 1 μl of 25 mM of dNTPs mix PBL) and RNAse free water. The thermal condition was 60 min at 42°C and after that; the reactions were heated to 95°C for 5 min in order to inactivate the enzyme.

### Polymerase Chain Reaction and Sequencing

The 5′-UTR of BVDV was amplified using “324–326” primers (17), while the 5′-UTR of HoBi Virus was amplified using “H1-326” primers (“H1”: 5′ CTGAAGCCCTGAGTACGGGG 3′). “H1” primer was designed using Primer 3 Software (18). Samples were run in a thermocycler (SimpliAmp, Applied Biosystems) in quadruplicate (two runs with two duplicates per run), for 324–326 and for H1-326 PCRs. The reactions were carried in a 25 μl volume containing 600 ηM of each primer, 5 μl of 5X GoTaq Reaction Buffer (Promega), 4.5 μl of mM MgCl_2_(Promega), 1 μl of 25 mM dNTPs mix, 1 U of Taq DNA polymerase (Promega) and DEPC water. PCR conditions were as follows: initial denaturing at 95°C for 5 min followed by 35 cycles of denaturing at 94°C for 1 min, primer annealing at 55°C for 1 min, and elongation at 72°C for 1 min. The final elongation was extended to 7 min at 72°C. The PCR products were detected by electrophoresis in a 2.0% agarose gel, stained with Gel Stain (Transbionovo), and visualized under ultraviolet light.

To evaluate the detection limit of the PCRs, 10-fold dilutions of the reference strains BVDV 1-Singer, BVDV 2-VS253 (kindly provided by Dr. Odeón, INTA Balcarce, Buenos Aires) and HoBi-like virus 83/10 were made in MEM and RNA extracted from each dilution was assessed by RT-PCR.

The amplicons were purified from agarose gels using a commercial kit (GE, Illustra GFX PCR DNA and Gel Band Purification Kit). Amplicons were not cloned but sequenced directly in both directions, and all samples were tested in duplicate. Sequencing was performed by the Genomic Unit at CICVyA-INTA, Castelar.

### Phylogenetic Analysis

The sequences generated were compared to sequences deposited in GenBank database using the BLAST software (http://www.ncbi.nlm.nih.gov/BLAST). Sequences were trimmed and analyzed with Bioedit Software (19) to obtain 236 bp fragments, corresponding to HoBi-like and BVD viruses. The analyzed fragments correspond to nucleotides 138–374 (5-UTR) of the reference strain BVDV1a-NADL, accession number M31182. For phylogenetic analysis, sequences of representative strains of BVDV-1, BVDV-2, HoBi-like virus, and CSFV were downloaded from the NCBI GenBank database. Multiple sequence alignment was performed with Clustal W, 3 Version 1.8.3 (20). The dendrograms were obtained under Kimura distances method (21) and Neighbor Joining (22) using Mega program, version 4 (23); 1,000 resample of bootstrap were used. Trees were drawn with Dendroscope 2.7.4 (24). Nucleotide sequences of the strains obtained during this study have been submitted to the GenBank database; accession numbers are given in Table 1.

**Table 1:** Pestivirus-positive samples obtained in this work.

**Sample^a^**	**Collection date**	**Species**	**Accession^b^**
4	2014	BVDV-1b	MF120552
6	2014	BVDV-1a	MF120553
7	2014	BVDV-1b	MF120554
8	2014	BVDV-1b	MF120555
11	2014	BVDV-1b	MF120556
13	2014	BVDV-1b	MF120557
14	2014	BVDV-1b	MF120558
17	2014	BVDV-1b	MF120559
18	2014	BVDV-1b	MF120560
26	2014	BVDV-1b	MF120561
31	2014	BVDV-1b	MF120562
35	2014	BVDV-1b	MF120563
37	2014	HoBi-like virus	MK017821
38	2014	BVDV-1b	MF120564
42	2014	BVDV-1b	MF120565
45	2014	BVDV-1b	MF120566
46	2014	BVDV-1b	MF120567
47	2014	BVDV-1a	MF120568
50	2014	BVDV-1b	MF120569
53	2015	BVDV-1b	MF120570
54	2015	BVDV-1b	MF120571
56	2015	BVDV-1b	MF120572
57	2015	BVDV-1b	MF120573
64	2015	BVDV-1b	MF120574
65	2015	BVDV-1b	MF120575
66	2015	BVDV-1a	MF120576
67	2015	BVDV-1b	MF120577
70	2015	BVDV-1b	MF120578
71	2015	HoBi-like virus	MH992642
74	2015	BVDV-1b	MF120580
77	2015	BVDV-1b	MF120581
78	2015	BVDV-1b	MF120582
79	2015	BVDV-1a	MF120583
83	2015	BVDV-1b	MF120584
96	2015	HoBi-like virus	MH992643
101	2015	BVDV-2b	MF120585
106	2015	BVDV-2a	MF120586
107	2015	BVDV-1b	MF120587
117	2015	HoBi-like virus	MH992641
118	2015	BVDV-1b	MF120588
146	2015	BVDV-1b	MF120589
150	2015	BVDV-1b	MF120590
176	2016	BVDV-1a	MF120591
181	2016	BVDV-1b	MF120592
184	2016	BVDV-1a	MF120593
214	2016	BVDV-1b	MF120594
P1	2015	BVDV-1b	MH992638
P2	2015	BVDV-1b	MH992639
P3	2015	BVDV-1b	MH992640

a *Fetal bovine serum samples. Samples 4-214 were obtained from provider A and P1-P3 were obtained from provider B*.

b *NCBI-NIH Genbank Accession numbers*.

### Virus Isolation

Irradiated and non-irradiated FBS, which tested positive using the H1-326 PCR protocol (37, 71, 117, and 126), were subjected to virus isolation in MDBK cells in 6-well plates. Cells were grown in MEM supplemented with 2% FBS, which was free from neutralizing antibodies (Nabs) against BVDV/HoBi-like virus and BVDV/HoBi-like virus RNA (determined by virus neutralization assay and RT-PCR, respectively), and a penicillin/streptomycin/gentamicin antibiotic cocktail. Four passages of 4 days each were performed using 70% confluent, 24-h fresh cell monolayers, freeze-thawing at room temperature between each passage to release any intracellular virus. Plates were kept at 37°C with 5% CO_2_ and monitored for cytopathic effect (cpe) during the period. Since none of the samples showed cpe, three more passages were performed. Following the fourth passage, the medium was removed and monolayers were washed twice with cold PBS and fixed in cold acetone. Indirect immunofluorescence was used to detect the HoBi-like virus, using the monoclonal antibody (mAb) N-2 (25) combined with a Goat anti-mouse Ig FITC Conjugated (Bio-x cat M17A27). The N-2 mAb reacts with a wide range of pestivirus species, including HoBi-like isolates. The HoBi-like strains were titrated on MDBK monolayers in 96-well plates using quadruplicate 10-fold dilutions and titer was estimated using indirect immunofluorescence as described above, and the Reed and Muench method (26).

### Antigenic Cross Reactivity Assay

#### Vaccination of Guinea Pig

The antigenic pattern of the Hobi-like virus isolates obtained from the non-irradiated FBS was evaluated as described previously (16). Briefly, four guinea pigs (strain SSi: Al from the CICVyA farm) 12 weeks of age, ~500 g in weight were obtained from the animal care facilities of National Institute of Agricultural Technology (INTA). Animals were inoculated subcutaneously with oil based vaccines formulated with the supernatants of the 7th passage of each HoBi-like virus-positive FBS sample (sample number identification: 37, 71, 96, 117). Another two guinea pigs were obtained as positive and negative controls and were inoculated with either the HoBi-like reference strain 83/10 (Decaro N, Department of Veterinary Medicine of Bari, Italy) (*n* = 1) or MDBK-mock supernatant (*n* = 1), respectively.

For the formulation, all viruses were diluted to a final concentration of 1 × 10^7^ TCID_50_/ml and 200 μl aliquots were placed on 48-well-culture plates. The aliquots were exposed to continuous UVC light 30 cm beneath the longitudinal midpoint of a UVC lamp (TUV 30WG30T8, Philips) for 1 min for inactivation. Afterward, the aliquots were subjected to viral isolation to confirm full inactivation. Each guinea pig received 3 doses of 0.6 ml each, on days 0 and 15 and 30. Each dose contained the equivalent of 2.4 × 10^6^ TCID_50_ of virus prior to inactivation. Vaccines were prepared with ISA 50 adjuvant (Seppic) in a 60:40 relation (adjuvant: antigen solution). Blood extraction was conducted on days 0 and 60 days post inoculation (dpi) by cardiac puncture under anesthesia, following INTA's ethical committee of animal welfare (CICUAE) recommendations for animal welfare. After centrifugation at 5,000 g for 5 min, sera samples were obtained. Guinea pig handling, inoculation, and sample collection was done by trained personnel under the supervision of a veterinarian and in accordance with protocols approved by the CICUAE Protocol 34/2017.

#### Virus-Neutralization Assay

In order to analyze the cross-reactivity between the HoBi-like virus isolates obtained from the FBS samples and reference strains of BVDV, comparative VN assays were performed using the guinea pig sera with the following cp strains: Singer (BVDV-1a), 25366 (BVDV-1b), VS253 (BVDV-2), and 83/10 (HoBi-like virus). Briefly, the VN assays were performed in 96-well plates containing monolayers of MDBK cells grown to 80% confluence; briefly, for each serum sample, a 75 μl viral suspension that contained 100 TCID of HoBi-83/10 virus was mixed with 75 μl of heat inactivated (55°C for 30 min) diluted guinea pig sera and were incubated for 1 h. These mixtures were then seeded on the plates and incubated at 37°C in 5% CO_2_ for 72 h. Control wells without virus were used for each serum sample in order to control for toxicity. The guinea pig sera were tested in duplicate in 2-fold dilutions starting at 1:8 and each test was carried out three times. Dilution endpoints were obtained for all the pestiviruses by observing cpe in cell monolayers.

The mean titers and the standard deviations for each VN test were calculated. Reciprocals of the endpoint Nab titers were calculated using Reed and Muench method.

## Results

### Polymerase Chain Reaction and Phylogenetic Analysis

Forty-five out of 124 samples analyzed (36%) were positive using 324–326 PCR, and 4 (3.2%) FBS samples were positive using H1-326 PCR. None of them resulted positive to both PCRs. The limit of detection for the PCRs used was 10^2^ TCID_50_/ml for their corresponding BVDV and HoBi reference strains (data not shown). The 324–326 primers amplify the BVDV-1 and BVDV-2 reference strains but not the Hobi reference strain. The opposite pattern is obtained with H1-326 primers.

**Figure 2:**
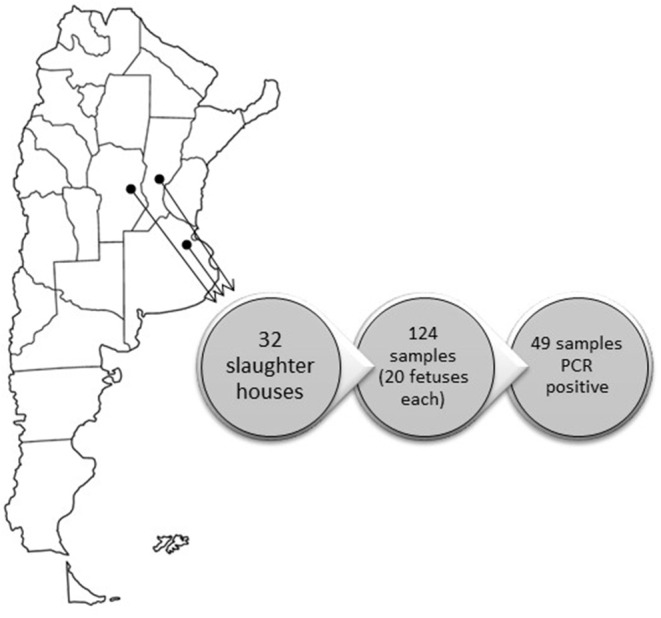
Phylogenetic analysis of Argentinean HoBi-isolates based on partial sequence of the 5′ Untranslated Region (UTR). The viral types and the Genbank accession numbers are indicated. The Argentinean strains are shown in italics and reference strains are highlighted. Branch lengths are proportional to genetic distances.

The phylogenetic analysis showed that the pestiviral RNA found in the FBS samples were segregated to three species, corresponding to BVDV-1 and BVDV-2 and HoBi-like virus strains (Table 1). Most of the 324–326 primer positive samples (43/45) grouped into the type 1 BVDV (88%). Of these type 1 BVDV strains, 86% (37/43) were grouped in subgenotype 1b cluster and 14% (6/43) clustered with 1a subgenotype reference strains (Figure 2). Of the two sequences clustered in BVDV-2 genotype, one belongs to subgenotype 2a (N°106) and the other to subgenotype 2b (N°101). The range of sequence identity among type 1a and 1b strains was: 97.6–100 and 94.3–100%, respectively, while among type 2 strains the values were 84.5–100%. The four samples positive to the H1-326 PCR were grouped in the Brazilian/Italian HoBi-like group (Figure 2). The sequence identity among them was 90.6–100%.

### Virus Isolation

The virus isolation of the four non-irradiated samples positive for HoBi-like virus by RT-PCR was achieved at the fourth passage in MDBK cells. Although three more passages were performed, none of the four viruses showed cpe. On the other hand, after the blind passages of the irradiated counter samples of the same FBS, immunofluorescence assay were negative, confirming the efficacy of the irradiation process.

### Antigenic Cross Reactivity Assay

The antigenic profiles of the isolated HoBi-like strains were checked in the guinea pig model. All the animals were seronegative to the strains evaluated at 0 dpi. After the second dose, the guinea pig vaccinated with the MDBK-mock supernatant, remained seronegative to the evaluated Pestiviruses. On the other hand, guinea pigs vaccinated with the different HoBi-like viruses developed Nabs against the reference HoBi-like strain (83/10), ranging from 128 to 512 at 60 dpi (Table 2).

**Table 2:** Neutralizing antibodies titers against different strains of BVDV and HoBi-like viruses in the sera of guinea pigs.

**Serum**	**HoBi 83/10**	**Nabs against**		
		**BVDV-1a**	**BVDV-1b**	**BVDV-2**
37	8.25 ± 0.4	Negative	Negative	Negative
71	7.5 ± 0.7	Negative	Negative	Negative
96	7.0 ± 0	Negative	Negative	5.5 ± 0.7
117	8.75 ± 0.4	Negative	Negative	5.75 ± 0.4
83/10 (C+)	10.6 ± 0	3.0 ± 0	3.75 ± 0,4	6.0 ± 0
Mock (C–)	Negative	Negative	Negative	Negative

*Antibody titers of guinea pigs at 60 dpi are indicated as mean values derived from three repetitive virus neutralization tests. 37, 71, 96, and 117 are the HoBi isolates obtained from the FBS samples used to vaccinate the guinea pigs. 83/10 is the reference cp Italian strain used to vaccinate a guinea pig (positive control), while Mock belongs to a supernatant of non-infected MDBK used to vaccinate another guinea pig (negative control). Animals that did not have neutralizing antibodies against the viruses evaluated in a 1/8 dilution were considered negative. Standard deviation is shown (±)*.

Further, cross neutralizing titers for BVDV-1a, BVDV-1b, and BVDV-2 were 4 to 6-fold lower than for the four HoBi-like isolates.

## Discussion

The presence of adventitious viruses in cell cultures is well-recognized as one of the main concerns in the manufacture of human and animal biological products. FBS is routinely used as a medium supplement and may harbor many viral agents, one of the most common being NCP-BVDV (27). The only previous study carried out to determine pestivirus contamination in Argentine FBS was published in 2000, which reported that 100 out of 200 (50%) FBS batches analyzed were BVDV-positive. Phylogenic characterization of detected strains of BVDV was not performed in that study (28). Multiple authors have reported different frequencies of BVDV contamination in FBS batches, ranging from 22 to 100% (8, 29–33). In most of these studies, the number of fetuses per batch was not available and, definitely, an increased pool size enhances the rate of contamination.

The FBS market in Argentina is led by a few national companies, which export their products to various countries including Brazil, Uruguay, Paraguay, Venezuela, Panama, USA, Kenya, and China. At the same time, other foreign companies commercialize their products in Argentina. Some national and foreign companies offer non-irradiated FBS, which has potential detrimental consequences, such as contamination of cell cultures used in basic research, virological diagnosis, upstream processing of biopharmaceuticals, embryo technology, and other animal reproduction protocols. A clear evidence of these risks is the frequent findings of viral agents' traces in cell lines, pharmaceutics products and vaccines (13).

Several methods to inactivate viruses in FBS have been evaluated (34). Gamma irradiation has been found to be effective while having minimum impact in the growth-promoting properties of sera. Still, it has been suggested that Gamma irradiation protocols need to be carefully evaluated as the complete inactivation will depend on various factors such as the serum packaging configuration, variability in gamma irradiation conditions and process, viral strain, viral load, variability in the quality of the virus stock selected for spiking studies, among others (35). In this regard, the results obtained in the model using lower values of gamma radiation (15 kGreys), according to the inactivation efficacies reported by Nims et al. (12) showed a dramatic variability in the results, based on the exponential behavior of the inactivation curve, increasing the risk from 1/19531 batches to 1 contaminated batch every 20 batches (15 kGrey). These results emphasize the need to strictly audit the industry standardization, monitoring, and quality assurance of inactivation protocols. Still, there are users who choose the non-irradiated variant of FBS alleging precipitation problems linked to the irradiation process. Anyway, we are convinced that the risk is of using non-irradiated FBS is too high and efforts to increase the awareness in the research community should be made. In addition, assessment of innovative inactivation protocols gives the chance to replace the gamma irradiation, while assuring the safety of the product.

Since the epidemiological status of the HoBi-like virus is still unknown in most countries and it has been reported a lack of sensitivity of the majority of the diagnostic techniques that are currently being used for this viral type, the potential risk of entry to a free-zone through FBS is significantly high. This fact makes urgently relevant the need to update and harmonize regulatory protocols for the commercialization of this product by regulatory authorities.

Because of the high rate of contamination of FBS with pestiviruses, sensitive monitoring for adventitious pestiviruses should be recommended to manufacturers and diagnostic laboratories, and be built into the framework of inspection controls on biological products and hemo-derivatives standards. Currently, no commercial diagnostic kits are licensed for the detection of HoBi-like virus, and at the same time, many techniques used *in-house* at diagnostic laboratories for the detection of pestivirus contamination have not been evaluated for their ability to detect HoBi-like virus. In this regard, although, molecular techniques for detection of HoBi pestiviruses have been reported (36, 37), its systematic implementation in diagnostic laboratories has been quite limited. Bauermann and collaborators (8) already suggested the urgent need for an update in pestivirus diagnostics and the use of multiple and complementary tests for an accurate result, otherwise, the prevalence of emerging HoBi-like pestivirus will remain being underestimated.

The regional prevalence of HoBi-like viruses is largely unknown. In the present report, most of the FBS samples belonged to Buenos Aires province (provider A), which presents the highest density of cattle in Argentina. In 4 of these samples, the detection of HoBi-like virus was achieved. It was not possible to trace the particular farm of origin, but this data indicates that HoBi-like virus is currently circulating in herds located in Pampean region. In the phylogenetic analysis, the local HoBi-like isolates obtained here were clustered within the Brazilian/Italian lineage (38). Although this result was expected due to the geographical location of Argentina and its proximity to Brazil, an introduction from a more distant region should not be dismissed.

It is only recently that the economic impact of the BVD in Argentina herds has begun to be analyzed (39, 40). Even less is known regarding the contribution of HoBi-like viruses to BVD losses. We believe that the results reported here justify the need to be proactive concerning the overhaul of diagnostic aspects for the certification of biological products to be commercialized and for import-export procedures.

The data obtained in this work affect not only the area of diagnosis but also the vaccine development sector. The antigenic variability between HoBi-like viruses and BVDV was demonstrated in the guinea-pig experiment (Table 2). However, there are no commercial vaccines, specifically against HoBi-like viruses, available (4, 38). It is critical these antigenic characteristics among bovine Pestiviruses to be taken into account by vaccine manufacturers, since it has been well-demonstrated that variability between vaccine and field strains may be cause of vaccine failure (41, 42). The results obtained regarding BVDV genotypes frequencies in the Pampean region indicate that BVDV-1b subgenotype may be, by far, the most common in the FBS samples, representing 75.5% of the whole number of BVDV- positive samples. This data reinforces one previous work, which indicated that 76.6% of the BVDV isolates from this region–obtained from years 1993 to 2010- belonged to BVDV-1b subgenotype (16). The incorporation of representative BVDV-1b field strains in national vaccines in order to provide more adequate protection against the disease was suggested in that study, but so far, there were no updates.

The high BVDV-1b prevalence is not the same throughout the continent, since other American countries (Uruguay, Brazil and Mexico) reported higher frequencies of the other BVDV variants (43, 44). However, it is relevant to highlight that in that previous study (16), all the analyzed samples belonged to clinical cases, fact that could have biased the results of prevalence of the described genotypes, due to differences in the pathogenicity generated by each strain. In the present work, that factor did not skew the results since the samples came from fetal samples obtained in the slaughterhouses and not from clinical cases. All the pestiviral RNA detected in this work correspond to viruses acting in natural infections and not to viruses derived from live or attenuated vaccines, since these formulations are not allowed in the country.

In conclusion, putting the procedures in place to assure the absence of pestiviruses as FBS contaminant is a critical part of the quality control of biological products or laboratory protocols. Therefore, diagnostic techniques used for the determination of pestiviruses in biological products should be frequently tested and updated for their ability to detect new variants of viruses.

## Data Availability Statement

Datasets are in a publicly accessible repository: The datasets generated for this study can be found in GenBank: https://www.ncbi.nlm.nih.gov/genbank/. The Genbank accession numbers are mentioned in the Table 1 of the manuscript.

## Ethics Statement

Animal handling, inoculation, and sample collection were performed by trained personnel under the supervision of a veterinarian and following national animal welfare regulations (Protocol No. 34/2017 from CICUAE, INTA).

## Author Contributions

AP carried out most of the experiments, with the collaboration of MP. AP and MP wrote the manuscript with strong support from JR. MD and JR procured funding and supervised the project.

### Conflict of Interest

JR was employed by company Ridpath Consulting. The remaining authors declare that the research was conducted in the absence of any commercial or financial relationships that could be construed as a potential conflict of interest.

## References

[1] Smith DB Meyers G Bukh J Gould EA Monath T Muerhoff AS. Proposed revision to the taxonomy of the genus Pestivirus, family Flaviviridae. J Gen Virol. (2017) 98:2106–12. 10.1099/jgv.0.000873 28786787 PMC5656787

[2] Weber MN Mósena ACS Simões SVD Almeida LL Pessoa CRM Budaszewski RF. Clinical presentation resembling mucosal disease associated with “HoBi”-like pestivirus in a field outbreak. Transbound Emerg Dis. (2014) 63:1–9. 10.1111/tbed.12223 24735072

[3] Shi H Kan Y Yao L Leng C Tang Q Ji J. Identification of natural infections in sheep/goats with HoBi-like pestiviruses in China. Transbound Emerg Dis. (2016) 63:480–4. 10.1111/tbed.12551 27478131

[4] Decaro N Mari V Sciarretta R Stella M Camero M Losurdo M. Comparison of the cross-antibody response induced in sheep by inactivated bovine viral diarrhoea virus 1 and Hobi-like pestivirus. Res Vet Sci. (2013) 94:806–8. 10.1016/j.rvsc.2012.11.016 23261155

[5] Decaro N Lucente MS Losurdo M Larocca V Elia G Occhiogrosso L. HoBi-like pestivirus and its impact on cattle productivity. Transbound Emerg Dis. (2016) 63:469–73. 10.1111/tbed.12529 27390140

[6] Decaro N Mari V Pinto P Lucente ME Sciarretta R Cirone F. Hobi-like pestivirus: both biotypes isolated from a diseased animal. J Gen Virol. (2012) 93:1976–83. 10.1099/vir.0.044552-0 22764319

[7] Decaro N Mari VM Lucente MS Sciarretta R Moreno A Armenise C. Experimental infection of cattle, sheep and pigs with ‘Hobi'-like pestivirus. Vet Mic. (2012) 155:165–71. 10.1016/j.vetmic.2011.08.030 21955447 PMC7126764

[8] Bauermann FV Flores EF Falkenberg SM Weiblen R Ridpath JF. Lack of evidence for the presence of emerging HoBi-like viruses in North American fetal bovine serum lots. J Vet Diagn Invest. (2014) 26:10–7. 10.1177/1040638713518208 24415196

[9] Silveira S Baumbach LF Weber MN Mósena ACS da Silva MS Cibulski SP. HoBi-like is the most prevalent ruminant pestivirus in Northeastern Brazil. Transbound Emerg Dis. (2017) 65:e113–20. 10.1111/tbed.12689 28758367

[10] Kozasa T Aoki H Nakajima N Fukusho A Ishimaru M Nakamura S. Methods to select suitable fetal bovine serum for use in quality control assays for the detection of adventitious viruses from biological products. Biologicals. (2011) 39:242–8. 10.1016/j.biologicals.2011.06.001 21719306

[11] Hawkes P Fetal bovine serum: geographic origin and regulatory relevance of viral contamination. Bioresour Bioprocess. (2015) 2:34 10.1186/s40643-015-0063-7

[12] Nims R Gauvin G Plavsic M. Gamma irradiation of animal sera for inactivation of viruses and mollicutes–a review. Biologicals. (2011) 39:370–7. 10.1016/j.biologicals.2011.05.003 21871817

[13] Laassri M Mee ET Connaughton SM Manukyan H Gruber M Rodriguez-hernandez C Detection of bovine viral diarrhoea virus nucleic acid, but not infectious virus, in bovine serum used for human vaccine manufacture. Biologicals. (2018) 55:63–70. 10.1016/j.biologicals.2018.06.002 29941334

[14] Pecora A Pérez Aguirreburualde MS Malacari D Zabal O Bauermann FV Ridpath JF Pestivirus emergentes HoBi: impacto en salud animal y su importancia como contaminante de insumos biotecnológicos. RIA. (2016) 42:252–7.

[15] Pecora A Pérez Aguirreburualde MS Malacari D Zabal O Sala JM Konrad JL. Serologic evidence of HoBi-like virus circulation in Argentinean water buffalo. J Vet Diagn Invest. (2017) 29:926–9. 10.1177/1040638717720246 28677409

[16] Pecora A Malacari D Ridpath JF Pérez Aguirreburualde MS Combessies G Odeón AC. First finding of genetic and antigenic diversity in 1b-BVDV isolates from Argentina. Res Vet Sci. (2014) 96:204–12. 10.1016/j.rvsc.2013.11.004 24295740

[17] Vilcek S Herring AJ Herring JA Nettleton PF Lowings JP Paton DJ. Pestiviruses isolated from pigs, cattle and sheep can be allocated into at least three genogroups using polymerase chain reaction and restriction endonuclease analysis. Arch Virol. (1994) 136:309–23. 10.1007/BF01321060 8031236

[18] Rozen S Skaletsky H. Primer3 on the WWW for general users and for biologist programmers. Methods Mol Biol. (2000) 132:365–86. 10.1385/1-59259-192-2:365 10547847

[19] Hall TA BioEdit: a user-friendly biological sequence alignment editor and analysis program for Windows 95/98/NT. In: Nucleic Acids Symposium Series No. 41 (Oxford) (1999). p. 95–8.

[20] Thompson JD Higgins DG Gibson TJ. CLUSTAL W: improving the sensitivity of progressive multiple sequence alignment through sequence weighting, position-specific gap penalties and weight matrix choice. Nucleic Acid Res. (1994) 22:4673–80. 10.1093/nar/22.22.4673 7984417 PMC308517

[21] Kimura M. A simple method for estimating evolutionary rates of base substitutions through comparative studies of nucleotide sequences. J Mol Evol. (1980) 16:111–20. 10.1007/BF01731581 7463489

[22] Saitou N Nei M. The neighbor-joining method: a new method for reconstructing phylogenetic trees. Mol Biol Evol. (1987) 4:406–25. Retrieved from: http://www.scopus.com/inward/record.url?eid=2-s2.0-0023375195&partnerID=tZOtx3y1 (accessed July 10, 2019). 3447015 10.1093/oxfordjournals.molbev.a040454

[23] Tamura K Dudley J Nei M Kumar S. MEGA4: molecular evolutionary genetics analysis (MEGA) software version 4.0. Mol Biol Evol. (2007) 24:1596–9. 10.1093/molbev/msm092 17488738

[24] Huson DH Richter DC Rausch C Dezulian T Franz M Rupp R. Dendroscope: an interactive viewer for large phylogenetic trees. BMC Bioinform. (2007) 8:460. 10.1186/1471-2105-8-460 18034891 PMC2216043

[25] Bauermann FV Falkenberg SM Vander Ley B Decaro N Brodersen BW Harmon A. Generation of calves persistently infected with HoBi-like pestivirus and comparison of methods for detection of these persistent infections. J Clin Microbiol. (2014) 52:3845–52. 10.1128/JCM.01563-14 25122860 PMC4313213

[26] Reed JL Muench H A simple method of estimating fifty percent endpoints. Am J Epidemiol. (1938) 27:493–7. 10.1093/oxfordjournals.aje.a118408

[27] Giangaspero M Pestivirus species potential adventitious contaminants of biological products. Trop Med Surg. (2013) 1:1–4. 10.4172/2329-9088.1000153

[28] Zabal O Kobrak A Lager IA Schudel AA Weber EL. Contamination of bovine fetal serum with bovine viral diarrhea virus. Rev Argent Microbiol. (2000) 32:27–32. 10785940

[29] Giammarioli M Ridpath JF Rossi E Bazzucchi M Casciari C De Mia GM. Genetic detection and characterization of emerging HoBi-like viruses in archival foetal bovine serum batches. Biologicals. (2015) 43:220–4. 10.1016/j.biologicals.2015.05.009 26071653

[30] Ridpath JF Bolin SR. Differentiation of types 1a, 1b and 2 bovine viral diarrhoea virus (BVDV) by PCR. Mol Cell Probes. (1998) 12:101–6. 10.1006/mcpr.1998.0158 9633045

[31] Yanagi M Bukh J Emerson SU Purcell RH. Contamination of commercially available fetal bovine sera with bovine viral diarrhea virus genomes: implications for the study of hepatitis C virus in cell cultures. J Infect Dis. (1996) 174:1324–7. 10.1093/infdis/174.6.1324 8940226

[32] Xia H Vijayaraghavan B Belak S Liu L. Detection and identification of the atypical bovine pestiviruses in commercial foetal bovine serum batches. PLoS ONE. (2011) 6:e28553. 10.1371/journal.pone.0028553 22174836 PMC3234271

[33] Toohey-Kurth K Sibley SD Goldberg TL. Metagenomic assessment of adventitious viruses in commercial bovine sera. Biologicals. (2017) 47:64–8. 10.1016/j.biologicals.2016.10.009 28366627

[34] Plavsic M Daley J Danner D Weppner D. Gamma irradiation of bovine sera. Dev Biol Stand. (1999) 99:95–109. 10404881

[35] Siegel W Fetal bovine serum: risk management. Bioprocess J. (2016) 15:22–5. 10.12665/J151.Siegel

[36] Losurdo M Mari V Lucentea MS Colaiannib ML Padalinob I Cavaliereb N. Development of a TaqMan assay for sensitive detection of all pestiviruses infecting cattle, including the emerging HoBi-like strains. J Virol Methods. (2015) 224:77–82. 10.1016/j.jviromet.2015.08.013 26300370 PMC7113749

[37] Mari V Losurdo M Lucente MS Lorusso E Elia G Martella V. Multiplex real-time RT-PCR assay for bovine viral diarrhea virus type, 1, type 2 and HoBi-like pestivirus. J Virol Methods. (2015) 229:1–7. 10.1016/j.jviromet.2015.12.003 26709100 PMC7113868

[38] Bauermann FV Ridpath JF Weiblen R Flores EF. HoBi-like viruses: an emerging group of pestiviruses. J Vet Diagn Invest. (2013) 25:6–15. 10.1177/1040638712473103 23345268

[39] Odeón AC González Altamiranda E Goizueta M Verna A Louge Uriarte E Spetter M Valorización económica de la implementación de una estrategia sanitaria de control del virus de la Diarrea Viral Bovina en un establecimiento de cria. Anales ANAV. (2015) 169:1–11.

[40] Sabatini D Santángelo F Pacífico C Cuantifican pérdidas económicas por enfermedades reproductivas. In: Seminario Sustentabilidad y modernización de la ganaderia Argentina (Vol. GEA) (Buenos Aires) (2013).

[41] Littledike ET Bolin SR Ridpath JF Consequences of Antigenic Diversity of Bovine Viral Diarrhea Virus. Nebraska City, NE: University of Nebraska (1993).

[42] Ridpath JF Neill JD. Pestiviruses: old enemies and new challenges. Anim Health Res Rev. (2015) 16:1–3. 10.1017/S1466252315000134 26050566

[43] Maya L Puentes R Reolón E Acuña P Riet F Rivero R. Molecular diversity of bovine viral diarrhea virus in uruguay. Arch Virol. (2016) 161:529–35. 10.1007/s00705-015-2688-4 26597189

[44] Pizarro-Lucero J Celedón MO Aguilera M de Calisto A. Molecular characterization of pestiviruses isolated from bovines in Chile. Vet Microbiol. (2006) 115:208–17. 10.1016/j.vetmic.2006.02.009 16563664

